# Animal models of migraine and experimental techniques used to examine trigeminal sensory processing

**DOI:** 10.1186/s10194-019-1043-7

**Published:** 2019-08-29

**Authors:** Andrea M. Harriott, Lauren C. Strother, Marta Vila-Pueyo, Philip R. Holland

**Affiliations:** 1 Neurovascular Research Lab, Department of Radiology, Massachusetts General Hospital, Charlestown, MA USA; 2 Department of Neurology, Massachusetts General Hospital, Boston, MA USA; 3 Headache Group, Department of Basic and Clinical Neuroscience, Institute of Psychology, Psychiatry and Neuroscience, King’s College London, James Black Centre, 125 Coldharbour Lane, London, SE5 9NU UK

**Keywords:** Pain, Migraine, Headache, Preclinical, Animal models, Electrophysiology, Translation

## Abstract

**Background:**

Migraine is a common debilitating condition whose main attributes are severe recurrent headaches with accompanying sensitivity to light and sound, nausea and vomiting. Migraine-related pain is a major cause of its accompanying disability and can encumber almost every aspect of daily life.

**Main body:**

Advancements in our understanding of the neurobiology of migraine headache have come in large from basic science research utilizing small animal models of migraine-related pain. In this current review, we aim to describe several commonly utilized preclinical models of migraine. We will discuss the diverse array of methodologies for triggering and measuring migraine-related pain phenotypes and highlight briefly specific advantages and limitations therein. Finally, we will address potential future challenges/opportunities to refine existing and develop novel preclinical models of migraine that move beyond migraine-related pain and expand into alternate migraine-related phenotypes.

**Conclusion:**

Several well validated animal models of pain relevant for headache exist, the researcher should consider the advantages and limitations of each model before selecting the most appropriate to answer the specific research question. Further, we should continually strive to refine existing and generate new animal and non-animal models that have the ability to advance our understanding of head pain as well as non-pain symptoms of primary headache disorders.

## Background

**Fig. 1:**
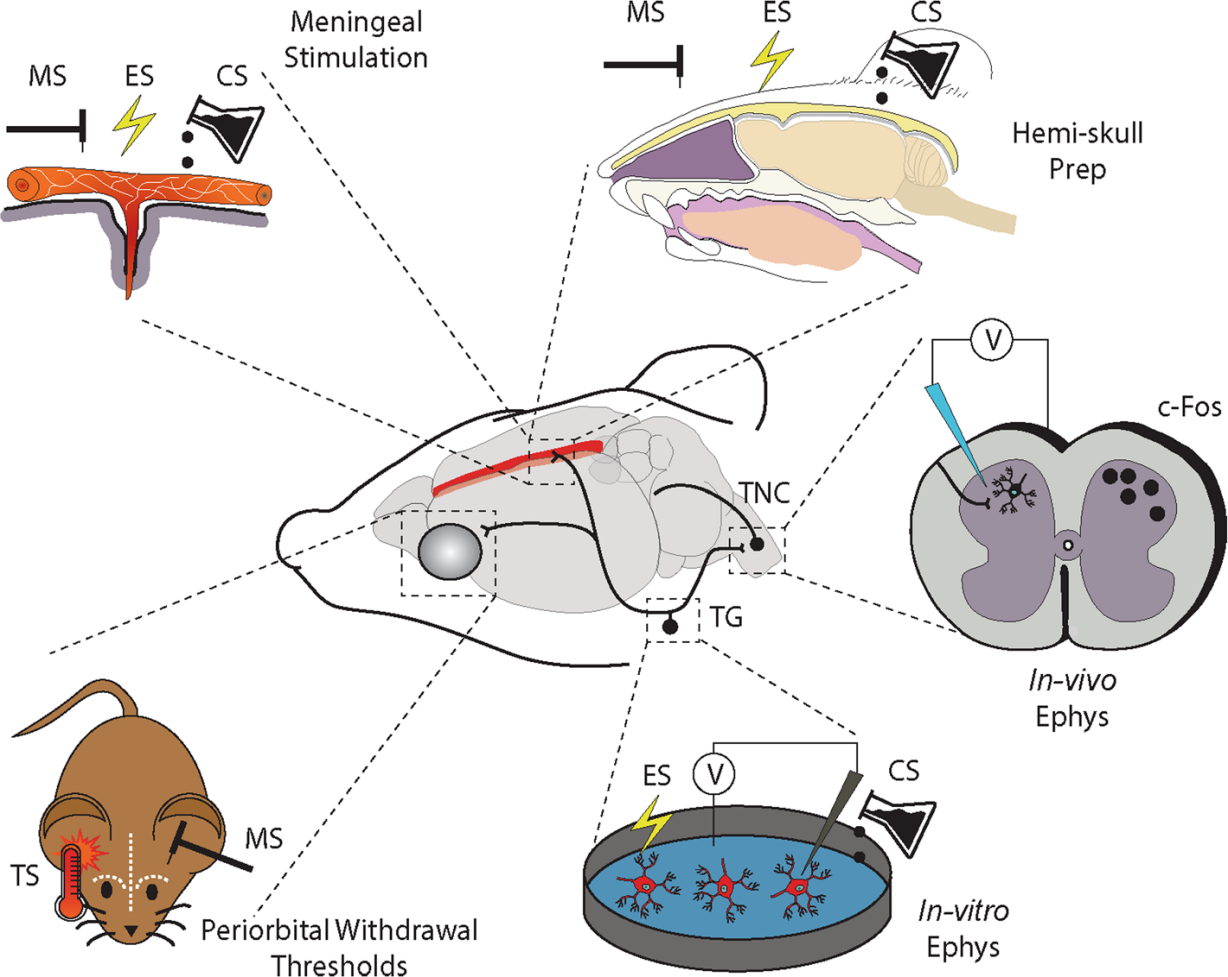
Selected models for assessing trigeminal sensory processing relevant to headache. Trigeminal afferents arising in the trigeminal ganglion (TG) convey sensory information from the intracranial and extracranial vasculature to the trigeminal nucleus caudalis (TNC). Second-order ascending projections then relay this information to the thalamus in combination with projections to key brainstem and hypothalamic nuclei. This pathway can be activated via mechanical (MS), electrical (ES) or chemical (CS) stimuli. Evoked activation of the trigeminovascular system can then be recorded using a variety of methodologies including, in-vivo electrophysiology (in-vivo Ephys) or markers of neuronal activation (e.g. c-Fos) in the TNC or alternate brain regions. A trigeminal ganglion-skull cavity in-vitro preparation (Hemi-skull Prep) has also been developed to preserve some degree of trigeminal/meningeal interface. Alternatively, trigeminal afferents arising in the TG can be dissociated and studied in-vitro using in-vitro electrophysiology (In-vitro Ephys). Finally, periorbital mechanical and thermal (TS) withdrawal thresholds can be assessed in the conscious behaving rodent to model more accurately altered pain responses at the whole animal level

Migraine is a debilitating condition whose main attributes are severe recurrent headaches with accompanying sensitivity to light and sound, nausea and vomiting. It is a highly prevalent and heterogeneous neurological disorder affecting approximately 6% of men and 18% of women [1] and is mediated by a combination of genetic [2] and environmental factors [3]. The pain associated with migraine is a major cause of its accompanying disability and can incumber almost every aspect of daily living [4, 5]. The disability associated with migraine underscores the need for selective and effective therapeutic tools. To that end, advancements in the neurobiology of migraine headache have come, in large part, from basic science research utilizing small animal models of migraine-related pain [6, 7] (Fig. 1). The recent development of new antibody drugs to treat migraine pain [8, 9] and pipeline therapies in development [10] is a testament to the translational potential of the animal models of migraine. Despite this, the complexity of migraine has been an impediment to fully modeling the disorder in animals and remains a major hurdle to overcome.

Several lines of evidence suggest that activation of trigeminal nociceptors innervating meningeal tissues including dural arteries and sinuses is central to the initiation of migraine pain [11]. Stimulation of these trigeminovascular afferents in humans can reproduce referred pain with qualitative similarity to migraine in cephalic regions [12]. While the central mechanisms by which trigeminovascular afferents are activated remains ill defined, once activated, they likely release neuropeptides including calcitonin gene related peptide (CGRP), substance P, neurokinin A and pituitary adenylate cyclase activating peptide (PACAP) in the dura, and centrally in the brainstem [13–15]. Release of CGRP peripherally from nociceptive afferents may mediate additional release of mast cell contents and other immune mediators [16]. Subsequent sensitization of trigeminovascular afferents and central sensitization of second order neurons in the trigeminal nucleus caudalis [17] are important component parts of migraine neurobiology that animal models ultimately seek to reproduce [11]. In this review, we aim to describe the current array of preclinical models available to researchers in the field. The diverse array of methodologies for triggering and measuring migraine-related pain phenotypes will be discussed and we will briefly highlight potential novel technologies and genetic tools that we feel will have a significant impact on preclinical migraine research in the next decade. We also highlight some clinical correlates to the models being studied, their emergence from clinical observation, potential in therapeutic testing and the extrapolation of their findings to the human experience of migraine. Animal models of migraine focused on trigeminal sensory processing have increased our mechanistic understanding of migraine pathobiology and have direct implications for target identification and translational research. Data from the models described has led to advances in drug therapy and a better comprehension of the mechanisms of drugs and devices currently approved for the treatment of migraine. Lastly, use of these models increased our understanding of the complex interplay of sex, environment and genetic influences on pain processing and migraine pathobiology. In addition to the following discussion, the reader is directed to several highly relevant review articles that complement the content herein [7, 18–22].

## Modeling migraine pain in the animal - experimental approaches

### In-vitro application of inflammatory mediators to dissociated meningeal afferents

Release of algogenic and inflammatory substances including nitric oxide, CGRP, neurokinin A, substance P, prostaglandins, and cytokines in the meninges are thought to influence the activation of trigeminovascular afferents [13, 23–25]. Moreover, in addition to the abundance of neuropeptide containing afferent terminals [26–29] meningeal tissues have a rich supply of mast cells. Mast cell degranulation may increase meningeal concentrations of histamine, serotonin and bradykinin further impacting trigeminovascular afferents [30–32].

The mechanisms of this trigeminovascular afferent activation can be studied in-vitro using application of these inflammatory substances to acutely dissociated or cultured trigeminal neurons. This includes targeted approaches using retrograde tracers from the dura mater to ensure the selection of acutely dissociated meningeal afferents. Electrophysiology techniques can then be utilized to examine changes specific for meningeal afferent excitability [33], ion channel modulation [34, 35] and afferent responses to current or novel migraine therapeutic targets [34], in the presence and absence of inflammatory mediators. For example, results from this model support inflammatory mediator-induced sensitization of trigeminovascular afferent nerve terminals via increases in tetrodotoxin resistant sodium currents, decreases in calcium dependent potassium currents, activation of a calcium dependent chloride current and increases in intracellular calcium signaling [35]. Furthermore, data from this model provided evidence that sumatriptan, a 5-HT_1B/1D_ receptor agonist and migraine abortive drug, can inhibit voltage-gated calcium currents and produce hyperpolarizing shifts in voltage-gated potassium currents in meningeal afferents [34]. Importantly, given the gender bias in migraine, specific sexually dimorphic responses have also been noted. Specifically, there was a greater proportion of dural afferents sensitized by inflammatory mediators in female as compared to male rats which may reflect sex-differences in activation of intracellular second-messenger pathways. There were also sex differences in active electrophysiological properties of the action potential waveform in females as compared to males following inflammatory mediator exposure suggesting differential inflammation induced modulation of voltage gated ion channels [36]. Additionally, in-vitro trigeminal neuron cultures can be combined with genetically modified animals (see Future Perspectives section) to examine changes more specific to migraine pathobiology. For example, trigeminal ganglion neurons isolated from mice expressing genetic mutations responsible for familial hemiplegic migraine (FHM) type 1 (R192Q mutation of the *CACNA1A* gene) were used to examine calcium/calmodulin dependent protein kinase II mediated increases in purinergic signaling [37].

Despite these important observations and the high-throughput nature of this model, there are several limitations that need to be carefully considered. The acute dissociation of ganglion neurons augments the distribution of proteins in the membrane and may cause some nerve injury and alterations in neuronal excitability, with important implications for migraine biology [38]. Additionally, the isolated nature of this model that can provide excellent mechanistic focus on meningeal afferents, also precludes the ability to study the interaction with other neuronal and non-neuronal populations including sympathetic and parasympathetic neurons and ganglionic satellite glial cells which may obfuscate extrapolation of the data obtained with this in-vitro model to the in-vivo environment. While such limitations are somewhat addressed in the adapted trigeminal ganglion-skull cavity in-vitro preparation (hemi-skull preparation) that attempts to preserve the hemi-dura, studies of meningeal inflammation are difficult. Nonetheless, using the in-vitro hemi-dura preparation, investigators were able to show that electrical stimulation of the trigeminal ganglion and application of inflammatory mediators on the dura increased meningeal CGRP release and produced longer-lasting increases in prostaglandin E2 [39]. Furthermore, in-vitro techniques are not commonly used to examine chronic or repetitive application of inflammatory substances over time. Therefore, the recurrent nature of migraine cannot be studied using this model. The observed findings, however, form an important basis for the determination of potential novel mechanisms in migraine-related pain and the in-vitro nature of the model has important ethical advantages, allowing relatively high throughput screening combined with a potential reduction in animal use [40].

### Direct electrical stimulation of trigeminal neurons in-vivo

There are currently three principal migraine models used to directly stimulate trigeminal neurons in-vivo. These models have been improved overtime to lessen their invasiveness and allow for chronic experimentation [18]. The first involves electrical stimulation of the trigeminal ganglion, the second electrical stimulation of the meningeal nerve terminal and the third chemical stimulation of meningeal afferent nerve terminals (see Administration of inflammatory substances to the meninges in-vivo section).

Firstly, the trigeminal ganglion of anesthetized animals can be electrically stimulated using inserted stereotactic bipolar electrodes. Trigeminal ganglion neurons are then activated using low frequency ( ~ 5-Hz) stimulation [41–43]. The benefit of this model is that tissue specific changes in the meninges and activation of central neurons and their response to drug therapy can be examined more directly as compared to in-vitro models. For example, data from this model has demonstrated that trigeminal ganglion stimulation causes release of CGRP from perivascular afferent terminals innervating the meninges. This release was accompanied by triptan sensitive ultrastructural morphometric changes in the neuropeptide containing nerve terminal swellings [41, 42]. While trigeminal ganglion electrical stimulation also produces activation of neurons in the trigeminal nucleus caudalis (as measured by expression of immediate early genes; see Immunohistochemistry section), this activation was not modulated by administration of sumatriptan [42]. While certain studies utilized prolonged stimulation paradigms (approximately 30 min) that may be considered supramaximal to induce morphological changes, shorter ganglionic stimulation (3–5 min) protocols also elicit peripheral neuropeptide release that is responsive to the triptans and dihydroergotamine [44]. The ability to target the trigeminal ganglion directly has many advantages, given its key role in the pathophysiology of migraine-related pain. Importantly, evidence using this approach support triptan and dihydroergotamine induced inhibition of peripheral neuropeptide release as a plausible mechanism of anti-nociceptive action [44]. However, the need to insert stimulating electrodes deep into the brain parenchyma has the potential to generate inflammatory responses both locally on the dura mater and throughout the central nervous regions traversed by the electrodes.

Secondly, similar to stimulation of the trigeminal ganglion, electrical stimulation of meningeal nerve terminals innervating the superior sagittal sinus [15], transverse sinus [45] or middle meningeal arteries [46, 47] to elicit trigeminal afferent activation have also been used to model migraine preclinically. These approaches evolved from the demonstration in humans: that their stimulation was considered painful and that this pain was often referred to the face [12] and that their stimulation in cats produced similar alterations in neuropeptide release to migraine patients [13, 15]. Moreover, direct stimulation of the intracranial vessels and subsequent activation of the meningeal afferents that innervate them, leads to the polysynaptic activation of the central projection sites of these afferents in the trigeminal nucleus caudalis and ascending projections throughout the central nervous system [48–51]. Such studies have proven critical in the ability to identify specific migraine-related pain processing nuclei throughout the brain. Furthermore, direct stimulation of nerve terminals innervating the intracranial vasculature and their meningeal afferents has proven a robust model to test differential responses to drug administration [45–47, 52, 53], similar to direct stimulation of the trigeminal ganglion as mentioned above [54, 55]. Importantly, this pharmacological testing has proven to be highly predictive of translational efficacy, both in terms of positive translation [46, 56, 57], therapeutic potential [58] and clinical trial failure [59], highlighting their continued utility. While in-vivo electrical stimulation models better account for the biological complexity of disease as compared to in-vitro models, they are limited by the invasiveness of craniotomy and tissue exposure. Additionally, upstream events leading to trigeminal activation are bypassed and stimulation parameters must be carefully regulated to prevent supramaximal stimulation that may not adequately represent a physiological state [18]. Animals are anesthetized and therefore different anesthetic regimes need to be considered. Lastly, while these models represent surrogate readouts of trigeminal nociceptive activation at the specific recording site, they do not incorporate many aspects of pain, or determine the overall pain phenotype at the whole animal level.

### Administration of inflammatory substances to the meninges in-vivo

Several experimental approaches use dural application of algogenic substances to model the proposed meningeal neurogenic inflammation thought to initiate migraine-related pain via trigeminovascular afferent and central neuronal sensitization [60, 61]. Inflammatory substances can be applied to the dura singly or in combination as an inflammatory soup. Commonly used substances include histamine, serotonin, bradykinin and prostaglandin E2. Other substances including capscaisin, low or high pH buffered solutions [62], cytokines [63] and complete Freund’s adjuvant [64, 65] have also been used. The application of these substances has been used to examine peripheral and central neuronal sensitization to various stimuli. Meningeal exposure to the above mentioned inflammatory substances alone or a combination as an inflammatory soup has been used as a reliable method of activating and sensitizing trigeminovascular meningeal afferents in-vivo as measured by enhanced trigeminal ganglion responses to mechanical stimulation of the meninges [61]. Meningeal application of this inflammatory soup also produces activation and sensitization of central neurons in the trigeminal nucleus with convergent dural and cutaneous receptive fields [60].

Over time, advancements in this model generated alternate delivery methods, requiring less invasive procedures that are now amenable to behavioral testing. While sensitivity of trigeminal ganglion and trigeminal nucleus caudalis neurons to mechanical stimulation following inflammatory soup infusion suggests heightened trigeminal nociception, assessment of conscious pain related reflexes in the animal offers an additional methodology for determining changes in pain perception. Response to mechanical stimulation using von Frey monofilament testing of the periorbital region in the awake behaving animal was therefore a critical evolution of the migraine pain model (see Behavior section). To permit behavioral testing in response to chemical dural stimulation, various models have been developed to allow for administration of substances in conscious behaving animals [66–69]. Repetitive inflammatory soup administration induces a chronic periorbital hypersensitivity to tactile stimuli that lasted for up to 3 weeks, suggestive of a model of chronic migraine [70]. In selecting this model, the researcher must consider that upstream events leading to trigeminal activation are bypassed and the chemical cocktail utilized requires careful control to prevent supramaximal stimulation. The surgical procedures, while improved, are intricate and could result in mast cell degranulation around the insertion site of the catheter.

### Exogenous administration of algogenic substances in-vivo

A key feature of migraine is that various triggers can initiate an attack and experimentally, chemical triggers have been used extensively in human models of migraine [71, 72]. Nitric oxide donors, including nitroglycerin, have emerged as the most prominent exogenous algogenic substances to date. This is based on early observations of their headache producing qualities in angina patients and during occupational exposure [73, 74]. However, more recently CGRP, PACAP and cilostazol have all emerged as viable human migraine triggers [75–77] and have been reverse translated into preclinical models of migraine pain [52, 58, 78, 79].

Importantly from a translational aspect, it is now becoming evident that such exogenous algogenic substances also trigger other migraine-related features in conjunction with pain responses. For example, both nitroglycerin and PACAP, but not CGRP, trigger migraine premonitory symptoms in patients [80–82] and nitroglycerin triggers cranial allodynia [52]. Recent preclinical studies have identified several pain-related and non-pain phenotypes following their administration and their utility is further enhanced by the ability to study both acute administration and a more chronic regime, considered relevant to migraine chronification [83].

The selection of a specific algogenic agent is dependent on the individual study requirements. Nitroglycerin and related nitric oxide donors have been used both in combination with in-vivo electrophysiological models where they induce a latent sensitization of trigeminal sensory afferents [52], and in freely behaving models where they induce increased activation of the trigeminovascular system and both periorbital and hindpaw hypersensitivity to tactile and thermal stimuli [84, 85]. As an alternate, based on the developing therapeutic potential of inhibiting CGRP signaling [8], the use of CGRP preclinically is increasing. Originally used in models of neurogenic dural vasodilation that explored therapeutic interventions on peripheral neurovascular CGRP signaling at the level of the dura mater [86, 87], more recently CGRP has been shown to trigger photophobia, periorbital hypersensitivity and spontaneous pain behaviours in rodents [79, 88, 89]. Furthermore, PACAP has shown preclinical potential. In the hemisected skull model, PACAP-38, but not PACAP-27 induced mast cell degranulation [90], while in-vivo studies demonstrate a delayed sensitization of trigeminovascular nociceptive processing [58] following PACAP infusion in rodents.

The use of algogenic, or migraine triggering agents in preclinical models of migraine-related pain has many advantages as well as important limitations to consider. The use of specific migraine triggering agents is strengthened by the specificity of such triggers, whereby alternate related molecules including vasoactive intestinal peptide (VIP), amylin or adrenomedullin, fail to induce pain behaviours or periorbital hypersensitivity in mice [88]. Importantly, exogenous algogenic substances are not restricted to specific peripheral or central nervous system sites and as such, have the potential to act more generally in migraine-relevant structures. This has clear advantages for disease modelling with the induction of premonitory symptoms in patients [80–82], however, it raises several unanswered questions regarding potential sites and mechanisms of action that need to be determined to fully appreciate their potential. Further, specific dosing regimens need to be adopted to allow a more thorough comparison between studies. For example, wild-type mice classically respond to a dose of 10 mg/kg of nitroglycerin, whereas mice harbouring genetic mutations linked to migraine with aura have been shown to respond to much lower doses [85]. Finally, such models have the potential to explore both acute responses and a more chronic state in rodents. Repetitive dosing over time has the potential to produce a prolonged basal hyperalgesia [83], however, such responses are also observed during chronic exposure to acute anti-migraine therapeutic agents [91], highlighting the complexity of determining divergent or shared downstream signaling cascades that may represent key targets for migraine.

## Experimental readouts: electrophysiology and immunohistochemistry

### Electrophysiology

As detailed previously, the activation of meningeal afferents that innervate the dural blood vessels, including the middle meningeal artery and the superior sagittal and transverse sinuses, results in headache pain that is very similar to the migrainous pain [12, 92]. Therefore, activation of the trigeminovascular system has consistently been used as a model of migraine-related pain. The trigeminovascular system includes the trigeminal ganglion, that sends primary sensory afferents to intra- and extracranial structures [93], including the dural blood vessels, and central projections to the trigeminal nucleus caudalis and the associated first and second cervical levels [94]. Second order neurons project from the trigeminal nucleus caudalis to higher order structures in the brainstem and diencephalic nuclei involved in pain processing [95–97]. As noted, several paradigms have been developed to facilitate the activation of the trigeminovascular system in-vivo and targeted electrode placement has facilitated the recording of durovascular evoked responses throughout the central nervous system. Given the importance of the meningeal afferents and their central synapses on the trigeminal nucleus caudalis, it is not surprising that the trigeminal nucleus caudalis has received considerable attention with respect to targeted neuronal activity recordings.

Electrophysiological recordings of trigeminal nucleus caudalis neuronal responses to nociceptive durovascular stimulation have been widely used as a readout of nociceptive trigeminovascular activation [45, 46, 98, 99]. The nature of the recordings from within the spinal cord dorsal horn necessitate the use of a laminectomy of the first cervical vertebrae along with an incision of the dura mater. Recording electrode placement is largely optimized via the mapping of cutaneous and dural receptive field responses, and once the appropriate level is located, specific cell types can be identified [18]. Second order trigeminothalamic projection neurons receive the majority of their inputs from thinly myelinated Aδ- and unmyelinated C-fibres [60, 100–103] arising in the trigeminal ganglion. As such, both fiber latency responses can be recorded and analyzed differentially to determine specific effects [104]. In addition to the specific A and C-fiber latencies, a variety of neuronal subtypes can be identified using high impedance electrodes that allow for single-cell responses to be recorded. The three major classes include low-threshold mechanoreceptors responding to innocuous stimulation, wide dynamic range neurons responding to both noxious and non-noxious stimuli, or nociceptive specific neurons that only respond to noxious input [105]. In addition to the trigeminal nucleus caudalis, in-vivo electrophysiology has the potential to map neuronal alterations in several migraine-relevant nuclei, with the thalamus also receiving considerable attention [106, 107] highlighting potential therapeutic benefits of modulating thalamocortical signaling.

In addition to the ability to target specific nuclei throughout the brain, direct trigeminal nucleus caudalis recording is commonly combined with alternate methodologies, including microinjection in discrete brain areas to discern functional connections. By combining these methodologies several modulatory networks regulating trigeminal nucleus caudalis durovascular evoked responses have been identified, including the A11 [108], the locus coeruleus [47], the ventrolateral periaqueductal gray [109, 110], thalamic [57] and hypothalamic [45] nuclei. Additionally, in seminal studies exploring the impact of environmental stimuli, potential mechanisms underlying light-induced exacerbation of durovascular nociceptive processing were identified in the posterior thalamus [111].

This in-vivo model has proven highly predictive in the pharmacological screening of potential anti-migraine compounds. Experimental pharmacological evidence has shown that effective treatments such as triptans [112–114], CGRP antibodies [104, 115], gepants [116], lasmiditan [117] and vagal nerve stimulation [118] all demonstrated significant efficacy; compounds that have failed clinical trials such as neurokinin 1 receptor antagonists do not [59].

A particular method to characterize the pharmacology of neuronal responses is the use of in-vivo electrophysiology in combination with microiontophoresis [119]. In this setup, a multi-barrel electrode, that includes a recording electrode and several capillaries, is used to pharmacologically modulate the neurons, that at the same time are being recorded by using the flow of electric charge through an aqueous solution to eject drugs to a small number of cells. Microiontophoretic ejection of ergot alkaloids [120] and triptans [121–123] in the trigeminal nucleus caudalis has been shown to inhibit nociceptive durovascular and local glutamate-evoked responses indicating a potential central action of these compounds. Interestingly, the later glutamatergic based direct activation may allow for a degree of site specificity to be defined, as the exogenous glutamate likely acts on postsynaptic receptors and thus an ability to selectively block this response would suggest a postsynaptic effect on trigeminothalamic projection neurons and not a direct effect on incoming meningeal afferents. This approach has identified potential central sites of action for several anti-migraine therapeutic targets including the CGRP receptor antagonist olcegepant [124] and the 5-HT_1B/1D_ receptor agonist naratriptan [107]. While the ability to determine potential local action of specific compounds is an advantage, it relies on bypassing the blood brain barrier that remains a significant barrier to the clinical development of central nervous system targets.

The use of in-vivo electrophysiology has several advantages, not least of which is the flexibility of methodologies to activate the underlying pathways of interest, including specific dural evoked responses and those elicited following the administration of exogenous algogenic substances. However, great care must be taken during surgical procedures and while modeling migraine-related pain in an intact nervous system with complex interfaces between the peripheral and central compartments is an advantage, the invasive nature of the surgery and subsequent disruption of the blood brain barrier must be carefully controlled for. Finally, current in-vivo electrophysiological procedures are largely acute in nature and therefore preclude longitudinal studies; future research should and most likely will take advantage of the increasing trend to conduct such studies in conscious behaving animals [125].

### Immunohistochemistry

#### C-Fos immunoreactivity

A complementary or alternate method to in-vivo models of migraine-related pain is the identification of neuronal activation in key nociceptive processing structures such as the trigeminal nucleus caudalis using markers of neuronal activation, mainly c-Fos immunoreactivity [126]. The gene FOS is an immediate early gene that encodes the proto-oncogene c-Fos, which dimerizes with transcription factors of the Jun family to build up the transcription factor AP-1 regulating the expression of downstream target genes [127]. In neurons, c-Fos expression can be stimulated by at least 3 second-messengers, including cAMP, protein kinase C and calcium-calmodulin, through the activation of the CREB/Cre complex [128]. c-Fos expression can be detected from 30 min to an hour after intense stimuli, reaching its peak at 2-4 h and returns to basal levels 8-24 h following stimulation [129]. Most commonly, c-Fos expression is visualized via the immunohistochemical detection of c-Fos in the nuceli of cell bodies [130].

c-Fos was one of the first transcription factors whose induction was shown to be activity-dependent [131]. Early studies identified that c-Fos is induced in the spinal dorsal horn following peripheral noxious stimulation [132], leading to its widespread use to study nociception [133]. In migraine-related pain research, c-Fos expression is commonly used as a valuable tool to identify subpopulations of neurons activated in response to noxious stimuli and related nociceptive pathways [19]. Hence, many studies have used c-Fos immunoreactivity to map neuronal activation throughout the trigeminovascular system, which has helped to generate a greater understanding of migraine pathophysiology [49, 113, 134–136]. Electrical, mechanical and chemical stimulation of meningeal afferents and systemic administration of algogenic substances including nitroglycerin induce c-Fos expression in the nociceptive-specific laminae of the trigeminal nucleus caudalis [49, 134, 137–139], which can be inhibited by anti-migraine treatments such as triptans [55, 84, 99, 140, 141], dihydroergotamine [99] and lasmiditan [142]. Thanks to the ability of c-Fos to respond to polysynaptic activation, this method also allows functional pathways to be mapped and therefore to determine ascending and descending pathways involved in migraine pathophysiology. To this end, c-Fos expression has been mapped in several brainstem structures, including the PAG [143–145], parabrachial nucleus and locus coeruleus [145]. Higher order diencephalic nuclei including the hypothalamus (e.g. the ventromedial nucleus, the supraoptic nucleus and the posterior hypothalamus [48, 51]) and thalamus (e.g. the thalamic reticular and centromedian nuclei [146, 147]).

The use of c-Fos expression has facilitated stepwise changes in our understanding of the pathophysiology of migraine and migraine-related pain. However, researchers must be wary of specific limitations, including the stimulus used to drive its expression [19]. This limitation is shown by the failure of substance P-neurokinin-1 receptor antagonists in acute and preventive treatment of migraine [148], although they have been shown to block c-Fos expression in the trigeminal nucleus caudalis following trigeminal ganglion stimulation [141, 149]. Importantly, the lack of c-Fos expression does not guarantee the absence of neuronal activation, as not all activated neurons express c-Fos, including those in the dorsal root ganglia [132]. Another important consideration is that the induction of quantifiable levels of c-Fos requires a strong consistent stimulation that is not usually physiologically relevant.

#### Alternative markers of neuronal activation

In certain conditions and with respect to specific tissues that do not express c-Fos, alternate markers of neuronal activation can prove beneficial. The extracellular signal-regulated kinase (ERK) is a member of the mitogen-activated protein kinase family. Once activated, phosphorylated ERK (pERK) is translocated into the nucleus where it activates several transcription factor s [150]. Like c-Fos, pERK expression [151] is very robust, requires high-threshold noxious stimuli and can be inhibited by analgesics. Unlike c-Fos, pERK expression is faster and more dynamic, it cannot be induced by innocuous stimuli and it is found in most subtype of neurons, including dorsal root ganglia neurons [152], as it is summarized in Table 1.

**Table 1:** Comparison of several features of c-Fos and pERK expression (Adapted from [152]). IHC, immunohistochemistry

	c-Fos	pERK
Methods of detection	IHC	IHC
Mechanism of detection	Gene expression	Phosphorylation
Induction by noxious stimuli	Yes	Yes
Induction by innocuous stimuli	Sometimes	No
Stimulus intensity-dependent	Yes	Yes
Subcellular distribution in neurons	Nucleus	Nucleus, cytoplasm, dendrites, axons
Time course after formalin injection		
Onset	30 min-1 h	1-3 min
Peak induction	1-2 h	2-10 min
Return to base	8-24 h	1-2 h

## Experimental readouts: behavioral assays

Measuring pain-like behaviours in awake, freely-behaving animals can provide key insights into the complex and integrative systems underlying migraine-like pain. An advantage of assessing pain-like behavior in conscious animals is the ability to assess the impact of experimental manipulations or therapeutic interventions on the whole animal. However, in doing so, it is imperative that the experimenter is blinded to treatments/experimental groups, as many behavioural assays can be subjective and therefore vulnerable to unconscious bias. When properly controlled, behavioural readouts are an invaluable tool for investigating migraine-like pain phenotypes underlying migraine pathophysiology.

Behavioural assays modeling migraine-like phenotypes can exploit sensory discriminative/evoked pain-like behaviors that focus on the trigeminal/spinal reflexes, operant models that assess cognitive aspects of pain, as well as spontaneous, non-evoked pain behavior. This section will focus on sensory discriminative readouts, as they are the most common and easily quantifiable, but will also touch briefly on other pain-like models as well.

### Modeling cutaneous allodynia

Sensory discriminative models in headache research tend to exploit a common associated symptom of migraine: cutaneous allodynia. Allodynia is defined as the perception of normal innocuous sensory stimuli as uncomfortable or painful. It has been reported that 70% of migraineurs experience cephalic allodynia: referred pain or sensitization around the head that is induced by the activation of the trigeminal system during an attack [153]; extracephalic allodynia in the arms and legs is reported in more severe and chronic cases [154, 155] and is likely attributed to sensitization of third order trigeminal neurons in the thalamus [153, 156]. As such, measurements of mechanical and thermal sensory nociceptive thresholds as a readout of cutaneous allodynia can be a reliable marker for migraine pathophysiology.

#### Mechanical allodynia

The most commonly used behavioural assessment of pain-like behavior in preclinical headache models is mechanical allodynia. Mechanical, or tactile, sensitivity is easily quantified by using calibrated von Frey filaments. These filaments are typically applied to the cephalic (whisker pad or periorbital areas) or extracephalic (hind paw) regions to determine evoked response thresholds. There are three widely used methods for how to apply the filaments and calculate a withdrawal response: the up-down method, ascending stimulus, and percent response rate. The up-down method calculates the threshold to illicit a response in 50% of the animals based on a statistical formula [157, 158]. This method involves applying the filaments in a pattern based on the animal’s response to the previous filament. If there was a positive response, the next filament applied would be the next one of less force; if there was a negative response, the next filament applied would be the one of next highest force. This would be repeated for five applications from the first positive response and a 50% mechanical withdrawal threshold calculated [157]. The ascending stimulus method sees filaments applied with increasing force until a withdrawal response is evoked and the force of this filament is recorded as the mechanical withdrawal threshold [159]. Percent response sees filaments of varying forces applied in ascending order 5–10 times and the number of positive responses to each filament are recorded and the percent response calculated [157, 160].

In preclinical headache research, allodynia is often assessed in response to dural inflammatory soup application or the administration of algogenic substances. As discussed previously, the most common and well established experimental migraine trigger is nitroglycerin. Preclinical studies commonly use nitroglycerin to sensitize the trigeminovascular system and a single dose (1-15 mg/kg) is known to induce mechanical allodynia that can last up to 4 h in rodents [83, 84, 88]. This increased sensitivity is therapeutically responsive to triptans [83, 84] and therefore strengthens this as a model of migraine-related pain. Preclinical investigations can thus utilize acute nitroglycerin administration to assess allodynia, investigate underlying mechanisms, or assess the efficacy of novel treatment targets by determining their ability to rescue nitroglycerin induced pain-related phenotypes. Transgenic mice, harboring a human mutation in casein-kinsase 1 delta, which is involved in regulating the molecular biological clock and has been linked to migraine in humans, have been shown to have altered hind paw mechanical sensitivity in response to nitroglycerin compared to controls [85]. As such, specific migraine-relevant genetic mutations appear to increase the sensitivity to nitroglycerin, which is in contrast to the previously identified inability of nitroglycerin to trigger attacks in familial hemiplegic migraine patients [161].

In addition to acute behavioural responses, repeated administration of nitroglycerin regimens have been established in order to assess biological mechanisms involved in migraine chronification. By repeated dosing, every other day for 9 days, a progressive and sustained basal hypersensitivity is observed in addition to the acute post treatment responses [83]. This basal hypersensitivity can be blocked by migraine preventives such as topiramate and propranolol [83, 162], supporting this as a model of chronic migraine and therefore be used to test novel anti-migraine preventatives. For example, ghrelin has been shown to attenuate nitroglycerin induced nociception by rescuing mechanical sensitivity, thus providing evidence that ghrelin has a modulating effect on central sensitization [163]. The basal hypersensitivity induced following chronic nitroglycerin is accompanied by increased CGRP expression in central brain areas with possible interaction with GABA and glutamate transmission that may contribute to the induction and maintenance of central sensitization [164]. Furthermore, direct stimulation of the nitric oxide receptor soluble guanylyl cyclase can chronically increase basal hypersensitivity which is subsequently blocked by acute and preventative migraine medications such as triptans and topiramate, thus indicating that nitroglycerin may in part cause migraine-related pain through stimulation of this pathway and that activation of this receptor may be an important component for the maintenance of chronic migraine [165].

In addition to nitroglycerin, other migraine provoking substances have been seen to elicit cephalic and extracephalic allodynic responses in rodents. Acute administration of CGRP, PACAP, histamine and prostaglandin E2 were shown to elicit periorbital mechanical sensitivity, which was attenuated by systemic antagonists [88]. Furthermore, intrathecal injection of CGRP has also been shown to induce hind paw mechanical allodynia in wild type mice and this response was further enhanced in transgenic mice that overexpress the CGRP receptor activity modifying protein 1 [166].

Finally, mechanical allodynia has been seen in response to trigeminal sensitization through other models such as inflammatory soup and cortical spreading depression. Application of inflammatory mediators onto the dura mater in awake, freely moving rats induces both facial and hind paw mechanical allodynia [67, 68, 70, 167], which is reversed by sumatriptan and CGRP receptor antagonist [67]. Cortical spreading depression, the electrophysiological correlate of migraine aura, can also activate trigeminal pain pathways. Cortical spreading depression induced mechanical allodynia has been observed in both the face and the hind paws of rats following multiple events [168]. Reduction in mechanical withdrawal thresholds ipsilateral to the cortical spreading depression can be reversed by a CGRP receptor antagonist [169].

#### Thermal allodynia

While most studies seem to assess mechanical allodynia, thermal allodynia (both hot and cold) can also be observed in preclinical models and can complement mechanical sensitivity to further dissect underlying mechanisms.

Cold sensitivity can be measured with ease in both cephalic and extracephalic body regions using the acetone evaporation test. Here, nociceptive behaviors are induced by the evaporative cooling of acetone on the skin and such behaviours can be counted, timed or scored [170, 171]. Assessing extracephalic thermal allodynia is easily assessed using the Hargreaves or tail flick test. The Hargreaves test involves directing a heat stimulus to the animals hind paw and measuring the withdrawal latency [172]. Additionally, a heat stimulus can be directed to the animals tail and withdrawal latency recorded. The heat stimulus can be in the form of an infrared beam or hot water bath (48 ± 5 °C). Using the Hargreaves assay, acute nitroglycerin [84] and chronic administration of algogenic substances increased thermal sensitivity [83, 165]. Furthermore, a different model of chronic migraine, nasocilary nerve ligation in rats, exhibits a lateralized sensitization to acetone following nitroglycerin in the forehead ipsilateral of nerve ligation [173]. One important differentiation is that behavioral studies have also shown differential response to mechanical and thermal allodynia, highlighting the complexity of trigeminal pain processing. For example, Brennan et al. showed that a higher dose of nitroglycerin was required to elicit differences in thermal allodynic responses in transgenic mice compared to a lower dose required for mechanical sensation [85]. In addition, Kim et al. showed differential mechanical and thermal sensitivities in the orofacial region and hind paw following chronic nitroglycerin [174]. In this study, cold was assessed on the face via acetone, while heat on the paw, likely due to the difficulty to direct a thermal stimulus to the orofacial region of an awake, freely moving animal.

One way this can be overcome is by the use of a novel operant behavioral assay using the orofacial pain assessment device (OPAD). Here, animals are trained to drink a reward while forced to place their face through temperature controlled thermal pads. Pain is assessed as a reduction in amount of reward consumed (quantified by number of licks) as well as contacts against the thermal pads [175, 176]. Recent work has shown that nitroglycerin treatment can decrease the amount of licks/contacts in wild type mice [177] indicating an increased sensitivity to thermal orofacial stimulation.

### Operant models for assessing cognitive aspects of pain

Operant pain assessment assays have been used in preclinical headache research to assess emotional dysfunction and affective-motivational components of pain. The advantage of operant based tests compared to other evoked sensory discrimination tests is that it is also a measure of higher order pain processing rather than relying on spinal reflex-based nociception. The orofacial pain assessment device mentioned above is also a readout of the emotional or motivational component of pain in that the animal needs to choose between a reward associated with a painful stimulus or forgoing the reward in order to avoid the pain [175], which adds translational value as it does not solely rely on the reflexive component of pain.

Another operant assay is the conditioned place aversion test, which measures the amount of time the animal spends in an area that has been associated with an aversive or painful stimulus. Chronic nitroglycerin has been shown to induce place avoidance, where animals learn to spend less time in the chamber associated with nitroglycerin and thus the painful experience. Novel therapeutic targets can prevent the condition place aversion either through analgesic effects or stimulating reward pathways [163, 178].

### Spontaneous pain behaviors

Spontaneous, or non-evoked behaviors can also be used as alternative readouts of pain and can be more indicative of headache-pain rather than the associated symptom of allodynia. Spontaneous behaviors such as exploration, locomotor activity, rearing or food and water consumption are thought to be general measures of a rodents overall wellbeing and can all decrease with pain. Other behaviors, such as freezing and grooming can increase and thus such behaviors can be measured as indirect markers of a pain-like state [179]. In migraine headache, activation of the trigeminovascular system can lead to headache worsened by activity, so freezing in rodents might reflect an activation of this system and a defense mechanism to restrict movement exacerbation of pain [169, 180]. Grooming reflects increased attention to the affected area, which may infer pain or discomfort. The above behaviors can be measured by observation or through a behavioral analysis system applied to a standard cage that is able to detect and classify behavioral variables based on vibrations produced by the movement of the animals. In headache research, it has been shown that cortical spreading depression can induce spontaneous pain behaviors such as freezing and grooming in freely moving rats and mice [169, 180, 181] which can subsequently be alleviated by a CGRP antagonist [169]. Additionally, trigeminal activation through the application of inflammatory mediators has also been shown to decrease activity and increase resting and grooming behavior which were then attenuated by a triptan [182].

## Future perspectives

While the above-mentioned models have significantly enhanced our understanding of migraine pathophysiology, led to the development of novel therapies and forged a path for future translational research in migraine; there remains an ongoing requirement to refine existing and generate novel models of migraine. While not covered here, such models should explore not only migraine-related pain, but attempt to explore alternate migraine-related phenotypes to better recapitulate the disorder as a whole.

### Advanced genetic modelling

Improvements in genome wide analysis studies have led to a wealth of data on polygenic risk factors for migraine with approximately 40 genetic loci identified [183]. This ever increasing list of risk factors is now combined with several rarer monogenic mutations responsible for specific migraine phenotypes [85, 184–186]. With advances in CRISPR/Cas9 technologies to facilitate gene editing in mice [187], the migraine field now has the potential to determine the specific impact of knocking in or out specific genes of interest. Such approaches have already proven effective. For example, generation of transgenic mice that overexpress the human receptor activity modifying protein 1 essential for the canonical CGRP receptor has facilitated several studies exploring CGRP hypersensitivity. These transgenic mice demonstrate clear nociceptive hypersensitivity to the algogenic substance CGRP combined with a photophobic phenotype suggesting a potential role for increased CGRP in the generation of photophobia [188, 189]. More recently a novel circadian related mutation resulting in the loss of function of casein kinase 1 delta and subsequent PERIOD-mediated phase advancing of the circadian clock has been identified [85]. In humans this mutation results in a familial advanced sleep phase and an extremely high penetrance of migraine with aura. Generation of a transgenic mouse that harbors the human mutation, enabled the identification of specific migraine phenotypes including a reduced threshold for triggering cortical spreading depression and an increased sensitivity to the human migraine trigger and algogenic substance nitroglycerin compared to wild type littermate mice. Such studies have the potential to enhance our knowledge on the impact of genetic and genetic/environment interaction’s on migraine susceptibility. In addition to specific gene editing approaches, an inbred rat model of spontaneous trigeminal allodynia has been described that is responsive to acute and preventive migraine therapies [190]. This model has recently been further inbred to generate a sustained trigeminal hypersensitivity that is responsive to acute migraine therapies with potential implications for modelling chronic migraine [191].

### Advanced viral vector approaches

The neuroscience field has experienced a vast expansion in the capability to selectively target specific neuronal populations using viral vector approaches. Such approaches allow for the targeted delivery and transfection of neurons based on their neurotransmitter profile, genetic makeup or anatomical distribution, while alternate transgenic mouse lines have been generated that specifically express chemogenetic and optogenetic constructs. While the use of such tools is in its infancy in migraine research, several studies have emerged that show the potential of such methodologies. For example, as discussed above, many current methods to activate the trigeminovascular system involve invasive cannula or electrode placement with potential impacts on cerebrovascular physiology. In a seminal study in the field, Houben et al. used optogenetic stimulation to activate channelrhodopsin-2 ion channels resulting in the activation of layer 5 cortical neurons and the subsequent induction of cortical spreading depression [192]. While this study primarily focused on the cortical spreading depression, it is appreciated that cortical spreading depression can act as a trigger to activate the trigeminovascular system [193] that is responsive to preventative migraine therapies [115] and trigger pain-related responses in rodents. As such, the combination of novel targeted optogenetic or chemogenetic approaches to evoke migraine-related pain and associated phenotypes in rodents holds enormous potential for the field. This approach is further enhanced by the ability to map detailed neuronal projections throughout the nervous system. More general tracing technologies have already been used to great effect in preclinical models of migraine-related pain including the exploration of pain processing pathways and potential photophobic and autonomic responses to light [194–197]. By utilizing novel tracing technologies that allow unrivaled precision down to the level of the single monosynaptic inputs on a specific cell type [198] the potential to map as yet unappreciated functional migraine-relevant brain networks holds significant potential.

## Conclusion

Our understanding of migraine-related pain processing and the development of novel therapeutics for its modulation has evolved via key translational research streams in humans and experimental animals. Herein, we have largely focused on the most commonly used preclinical models of migraine-related pain; however, despite significant success, there remains a major unmet need to generate novel knowledge on the underlying mechanisms of migraine initiation, associated symptoms and ultimately attack cessation. Future preclinical research is a necessity to achieve this goal, and while alternate readouts that can help to reduce the use of animals in research are encouraged, for now the whole animal is the only model that can accurately examine the complex interactions between the peripheral and central nervous systems. As such, it is essential that researchers continue to refine existing and develop novel models to enable studies that explore the diverse symptomatology of migraine, while continuing to achieve significant translational success in terms of novel disease modifying therapies.

## Data Availability

Not applicable.

## References

[1] Lipton RB (2001). Prevalence and burden of migraine in the United States: data from the American migraine study II. Headache.

[2] Gormley P (2018). Common variant burden contributes to the familial aggregation of migraine in 1,589 families. Neuron.

[3] Goadsby PJ (2017). Pathophysiology of migraine: a disorder of sensory processing. Physiol Rev.

[4] Agosti R (2018). Migraine burden of disease: from the Patient’s experience to a socio-economic view. Headache.

[5] G. B. D. Headache Collaborators (2018). Global, regional, and national burden of migraine and tension-type headache, 1990-2016: a systematic analysis for the global burden of disease study 2016. Lancet Neurol.

[6] Akerman S, Romero-Reyes M, Holland PR (2017). Current and novel insights into the neurophysiology of migraine and its implications for therapeutics. Pharmacol Ther.

[7] Vuralli D (2019). Behavioral and cognitive animal models in headache research. J Headache Pain.

[8] Goadsby PJ (2017). A controlled trial of Erenumab for episodic migraine. N Engl J Med.

[9] Silberstein SD (2017). Fremanezumab for the preventive treatment of chronic migraine. N Engl J Med.

[10] Goadsby Peter J, Wietecha Linda A, Dennehy Ellen B, Kuca Bernice, Case Michael G, Aurora Sheena K, Gaul Charly (2019). Phase 3 randomized, placebo-controlled, double-blind study of lasmiditan for acute treatment of migraine. Brain.

[11] Olesen J (2009). Origin of pain in migraine: evidence for peripheral sensitisation. Lancet Neurol.

[12] Ray BW, Wolff HG (1940). Experimental studies on headache*.* Pain sensitive structures of the head and their significance in headache. Arch Surg.

[13] Goadsby PJ, Edvinsson L (1993). The trigeminovascular system and migraine: studies characterizing cerebrovascular and neuropeptide changes seen in humans and cats. Ann Neurol.

[14] Noseda Rodrigo, Burstein Rami (2013). Migraine pathophysiology: Anatomy of the trigeminovascular pathway and associated neurological symptoms, cortical spreading depression, sensitization, and modulation of pain. Pain.

[15] Zagami AS, Goadsby PJ, Edvinsson L (1990). Stimulation of the superior sagittal sinus in the cat causes release of vasoactive peptides. Neuropeptides.

[16] Ottosson A, Edvinsson L (1997). Release of histamine from dural mast cells by substance P and calcitonin gene-related peptide. Cephalalgia.

[17] Yarnitsky D (2003). 2003 Wolff award: possible parasympathetic contributions to peripheral and central sensitization during migraine. Headache.

[18] Akerman S, Holland PR, Hoffmann J (2013). Pearls and pitfalls in experimental in vivo models of migraine: dural trigeminovascular nociception. Cephalalgia.

[19] Bergerot A (2006). Animal models of migraine: looking at the component parts of a complex disorder. Eur J Neurosci.

[20] Demartini C (2019). Nitroglycerin as a comparative experimental model of migraine pain: from animal to human and back. Prog Neurobiol.

[21] Munro G, Jansen-Olesen I, Olesen J (2017). Animal models of pain and migraine in drug discovery. Drug Discov Today.

[22] Pietrobon D, Brennan KC (2019). Genetic mouse models of migraine. J Headache Pain.

[23] Gallai V (1995). Vasoactive peptide levels in the plasma of young migraine patients with and without aura assessed both interictally and ictally. Cephalalgia.

[24] Sarchielli P (2006). Proinflammatory cytokines, adhesion molecules, and lymphocyte integrin expression in the internal jugular blood of migraine patients without aura assessed ictally. Headache.

[25] Sarchielli P (2000). Nitric oxide metabolites, prostaglandins and trigeminal vasoactive peptides in internal jugular vein blood during spontaneous migraine attacks. Cephalalgia.

[26] Edvinsson L (1988). Neurokinin a in cerebral vessels: characterization, localization and effects in vitro. Regul Pept.

[27] Uddman R, Edvinsson L (1989). Neuropeptides in the cerebral circulation. Cerebrovasc Brain Metab Rev.

[28] Uddman R (1985). Innervation of the feline cerebral vasculature by nerve fibers containing calcitonin gene-related peptide: trigeminal origin and co-existence with substance P. Neurosci Lett.

[29] Uddman R (1993). PACAP, a VIP-like peptide: immunohistochemical localization and effect upon cat pial arteries and cerebral blood flow. J Cereb Blood Flow Metab.

[30] Hassler SN (2019). Protease activated receptor 2 (PAR2) activation causes migraine-like pain behaviors in mice. Cephalalgia.

[31] Levy D (2007). Mast cell degranulation activates a pain pathway underlying migraine headache. Pain.

[32] Rozniecki JJ (1999). Morphological and functional demonstration of rat dura mater mast cell-neuron interactions in vitro and in vivo. Brain Res.

[33] Harriott AM, Gold MS (2009). Electrophysiological properties of dural afferents in the absence and presence of inflammatory mediators. J Neurophysiol.

[34] Harriott AM, Scheff NN, Gold MS (2012). The complex actions of sumatriptan on rat dural afferents. Cephalalgia.

[35] Vaughn AH, Gold MS (2010). Ionic mechanisms underlying inflammatory mediator-induced sensitization of dural afferents. J Neurosci.

[36] Scheff NN, Gold MS (2011). Sex differences in the inflammatory mediator-induced sensitization of dural afferents. J Neurophysiol.

[37] Nair A (2010). Familial hemiplegic migraine Ca(v)2.1 channel mutation R192Q enhances ATP-gated P2X3 receptor activity of mouse sensory ganglion neurons mediating trigeminal pain. Mol Pain.

[38] Huang ZJ (2012). Chronic compression or acute dissociation of dorsal root ganglion induces cAMP-dependent neuronal hyperexcitability through activation of PAR2. Pain.

[39] Ebersberger A (1999). Release of substance P, calcitonin gene-related peptide and prostaglandin E2 from rat dura mater encephali following electrical and chemical stimulation in vitro. Neuroscience.

[40] Kilkenny C (2010). Animal research: reporting in vivo experiments: the ARRIVE guidelines. Br J Pharmacol.

[41] Knyihar-Csillik E (1995). Electrical stimulation of the Gasserian ganglion induces structural alterations of calcitonin gene-related peptide-immunoreactive perivascular sensory nerve terminals in the rat cerebral dura mater: a possible model of migraine headache. Neurosci Lett.

[42] Knyihar-Csillik E (1997). Effect of a serotonin agonist (sumatriptan) on the peptidergic innervation of the rat cerebral dura mater and on the expression of c-fos in the caudal trigeminal nucleus in an experimental migraine model. J Neurosci Res.

[43] Limmroth V (2001). An in vivo rat model to study calcitonin gene related peptide release following activation of the trigeminal vascular system. Pain.

[44] Buzzi MG (1991). Dihydroergotamine and sumatriptan attenuate levels of CGRP in plasma in rat superior sagittal sinus during electrical stimulation of the trigeminal ganglion. Neuropharmacology.

[45] Robert C (2013). Paraventricular hypothalamic regulation of trigeminovascular mechanisms involved in headaches. J Neurosci.

[46] Holland PR (2012). Acid-sensing ion channel 1: a novel therapeutic target for migraine with aura. Ann Neurol.

[47] Vila-Pueyo M (2019). Divergent influences of the locus coeruleus on migraine pathophysiology. Pain.

[48] Benjamin L (2004). Hypothalamic activation after stimulation of the superior sagittal sinus in the cat: a Fos study. Neurobiol Dis.

[49] Kaube H (1993). Expression of c-Fos-like immunoreactivity in the caudal medulla and upper cervical spinal cord following stimulation of the superior sagittal sinus in the cat. Brain Res.

[50] Knight YE (2005). Patterns of fos expression in the rostral medulla and caudal pons evoked by noxious craniovascular stimulation and periaqueductal gray stimulation in the cat. Brain Res.

[51] Malick A (2001). A neurohistochemical blueprint for pain-induced loss of appetite. Proc Natl Acad Sci U S A.

[52] Akerman S (2019). Nitroglycerine triggers triptan-responsive cranial allodynia and trigeminal neuronal hypersensitivity. Brain.

[53] Goadsby PJ, Hoskin KL (1999). Differential effects of low dose CP122,288 and eletriptan on fos expression due to stimulation of the superior sagittal sinus in cat. Pain.

[54] Goadsby PJ, Edvinsson L (1994). Joint 1994 Wolff award presentation. Peripheral and central trigeminovascular activation in cat is blocked by the serotonin (5HT)-1D receptor agonist 311C90. Headache.

[55] Knyihar-Csillik E (2000). Effects of eletriptan on the peptidergic innervation of the cerebral dura mater and trigeminal ganglion, and on the expression of c-fos and c-Jun in the trigeminal complex of the rat in an experimental migraine model. Eur J Neurosci.

[56] Storer RJ, Goadsby PJ (2004). Topiramate inhibits trigeminovascular neurons in the cat. Cephalalgia.

[57] Summ O (2010). Modulation of nocioceptive transmission with calcitonin gene-related peptide receptor antagonists in the thalamus. Brain.

[58] Akerman S, Goadsby PJ (2015). Neuronal PAC1 receptors mediate delayed activation and sensitization of trigeminocervical neurons: Relevance to migraine. Sci Transl Med.

[59] Goadsby PJ, Hoskin KL, Knight YE (1998). Substance P blockade with the potent and centrally acting antagonist GR205171 does not effect central trigeminal activity with superior sagittal sinus stimulation. Neuroscience.

[60] Burstein R (1998). Chemical stimulation of the intracranial dura induces enhanced responses to facial stimulation in brain stem trigeminal neurons. J Neurophysiol.

[61] Strassman AM, Raymond SA, Burstein R (1996). Sensitization of meningeal sensory neurons and the origin of headaches. Nature.

[62] Avona A (2019). Dural calcitonin gene-related peptide produces female-specific responses in rodent migraine models. J Neurosci.

[63] Zhang X, Burstein R, Levy D (2012). Local action of the proinflammatory cytokines IL-1beta and IL-6 on intracranial meningeal nociceptors. Cephalalgia.

[64] Lukacs M (2015). Dural administration of inflammatory soup or complete Freund's adjuvant induces activation and inflammatory response in the rat trigeminal ganglion. J Headache Pain.

[65] Lukacs M (2017). Topical dura mater application of CFA induces enhanced expression of c-fos and glutamate in rat trigeminal nucleus caudalis: attenuated by KYNA derivate (SZR72). J Headache Pain.

[66] Burgos-Vega CC (2019). Non-invasive dural stimulation in mice: a novel preclinical model of migraine. Cephalalgia.

[67] Edelmayer RM (2009). Medullary pain facilitating neurons mediate allodynia in headache-related pain. Ann Neurol.

[68] Wieseler J (2010). A novel method for modeling facial allodynia associated with migraine in awake and freely moving rats. J Neurosci Methods.

[69] Wieseler J (2012). Indwelling supradural catheters for induction of facial allodynia: surgical procedures, application of inflammatory stimuli, and behavioral testing. Methods Mol Biol.

[70] Oshinsky ML, Gomonchareonsiri S (2007). Episodic dural stimulation in awake rats: a model for recurrent headache. Headache.

[71] Ashina M (2017). Human models of migraine - short-term pain for long-term gain. Nat Rev Neurol.

[72] Ashina M, Hansen JM, Olesen J (2013). Pearls and pitfalls in human pharmacological models of migraine: 30 years’ experience. Cephalalgia.

[73] McGuinness BW, Harris EL (1961). “Monday head”: an interesting occupational disorder. Br Med J.

[74] Murrell W (1879). Nitro-glycerine as a remedy for angina pectoris. Lancet.

[75] Guo S, Olesen J, Ashina M (2014). Phosphodiesterase 3 inhibitor cilostazol induces migraine-like attacks via cyclic AMP increase. Brain.

[76] Hansen JM (2010). Calcitonin gene-related peptide triggers migraine-like attacks in patients with migraine with aura. Cephalalgia.

[77] Schytz HW (2009). PACAP38 induces migraine-like attacks in patients with migraine without aura. Brain.

[78] Christensen SL (2018). Cilostazol induces C-fos expression in the trigeminal nucleus caudalis and behavioural changes suggestive of headache with the migraine-like feature photophobia in female rats. Cephalalgia.

[79] Rea BJ (2018). Peripherally administered calcitonin gene-related peptide induces spontaneous pain in mice: implications for migraine. Pain.

[80] Afridi SK, Kaube H, Goadsby PJ (2004). Glyceryl trinitrate triggers premonitory symptoms in migraineurs. Pain.

[81] Guo S (2016). Premonitory and nonheadache symptoms induced by CGRP and PACAP38 in patients with migraine. Pain.

[82] Maniyar FH (2014). Brain activations in the premonitory phase of nitroglycerin-triggered migraine attacks. Brain.

[83] Pradhan AA (2014). Characterization of a novel model of chronic migraine. Pain.

[84] Bates EA (2010). Sumatriptan alleviates nitroglycerin-induced mechanical and thermal allodynia in mice. Cephalalgia.

[85] Brennan KC (2013). Casein kinase idelta mutations in familial migraine and advanced sleep phase. Sci Transl Med.

[86] Holland PR, Akerman S, Goadsby PJ (2005). Orexin 1 receptor activation attenuates neurogenic dural vasodilation in an animal model of trigeminovascular nociception. J Pharmacol Exp Ther.

[87] Williamson DJ (1997). The novel anti-migraine agent rizatriptan inhibits neurogenic dural vasodilation and extravasation. Eur J Pharmacol.

[88] De Logu F (2019). Migraine-provoking substances evoke periorbital allodynia in mice. J Headache Pain.

[89] Mason BN (2017). Induction of migraine-like photophobic behavior in mice by both peripheral and central CGRP mechanisms. J Neurosci.

[90] Pedersen SH (2019). PACAP-38 and PACAP (6-38) Degranulate rat meningeal mast cells via the orphan MrgB3-receptor. Front Cell Neurosci.

[91] De Felice M (2010). Triptan-induced latent sensitization: a possible basis for medication overuse headache. Ann Neurol.

[92] Penfield WM, McNaughton FR (1940). Dural headache and innervation of the dura mater. Arch Neurol Psychiatr.

[93] Amin FM (2013). Magnetic resonance angiography of intracranial and extracranial arteries in patients with spontaneous migraine without aura: a cross-sectional study. Lancet Neurol.

[94] May A, Goadsby PJ (1999). The trigeminovascular system in humans: pathophysiologic implications for primary headache syndromes of the neural influences on the cerebral circulation. J Cereb Blood Flow Metab.

[95] Akerman S, Holland PR, Goadsby PJ (2011). Diencephalic and brainstem mechanisms in migraine. Nat Rev Neurosci.

[96] Liu Y (2009). Brainstem and thalamic projections from a craniovascular sensory nervous Centre in the rostral cervical spinal dorsal horn of rats. Cephalalgia.

[97] Matsushita M, Ikeda M, Okado N (1982). The cells of origin of the trigeminothalamic, trigeminospinal and trigeminocerebellar projections in the cat. Neuroscience.

[98] Akerman S, Holland PR, Goadsby PJ (2007). Cannabinoid (CB1) receptor activation inhibits trigeminovascular neurons. J Pharmacol Exp Ther.

[99] Hoskin KL, Kaube H, Goadsby PJ (1996). Central activation of the trigeminovascular pathway in the cat is inhibited by dihydroergotamine. A c-Fos and electrophysiological study. Brain.

[100] Holland PR, Akerman S, Goadsby PJ (2006). Modulation of nociceptive dural input to the trigeminal nucleus caudalis via activation of the orexin 1 receptor in the rat. Eur J Neurosci.

[101] Liu Y, Broman J, Edvinsson L (2004). Central projections of sensory innervation of the rat superior sagittal sinus. Neuroscience.

[102] Liu Y, Broman J, Edvinsson L (2008). Central projections of the sensory innervation of the rat middle meningeal artery. Brain Res.

[103] Millan MJ (2002). Descending control of pain. Prog Neurobiol.

[104] Melo-Carrillo A (2017). Fremanezumab-A Humanized Monoclonal Anti-CGRP Antibody-Inhibits Thinly Myelinated (Adelta) But Not Unmyelinated (C) Meningeal Nociceptors. J Neurosci.

[105] Hu J, Milenkovic N, Lewin GR (2006). The high threshold mechanotransducer: a status report. Pain.

[106] Andreou AP, Shields KG, Goadsby PJ (2010). GABA and valproate modulate trigeminovascular nociceptive transmission in the thalamus. Neurobiol Dis.

[107] Shields KG, Goadsby PJ (2006). Serotonin receptors modulate trigeminovascular responses in ventroposteromedial nucleus of thalamus: a migraine target?. Neurobiol Dis.

[108] Charbit AR, Akerman S, Goadsby PJ (2011). Trigeminocervical complex responses after lesioning dopaminergic A11 nucleus are modified by dopamine and serotonin mechanisms. Pain.

[109] Knight YE, Goadsby PJ (2001). The periaqueductal grey matter modulates trigeminovascular input: a role in migraine?. Neuroscience.

[110] Pozo-Rosich P (2015). Periaqueductal gray calcitonin gene-related peptide modulates trigeminovascular neurons. Cephalalgia.

[111] Noseda R (2016). Migraine photophobia originating in cone-driven retinal pathways. Brain.

[112] Goadsby PJ, Hoskin KL (1996). Inhibition of trigeminal neurons by intravenous administration of the serotonin (5HT)1B/D receptor agonist zolmitriptan (311C90): are brain stem sites therapeutic target in migraine?. Pain.

[113] Goadsby PJ, Hoskin KL (1997). The distribution of trigeminovascular afferents in the nonhuman primate brain *Macaca nemestrina*: a c-fos immunocytochemical study. J Anat.

[114] Kaube H, Hoskin KL, Goadsby PJ (1993). Inhibition by sumatriptan of central trigeminal neurones only after blood-brain barrier disruption. Br J Pharmacol.

[115] Melo-Carrillo A (2017). Selective Inhibition of Trigeminovascular Neurons by Fremanezumab: A Humanized Monoclonal Anti-CGRP Antibody. J Neurosci.

[116] Feistel S, Albrecht S, Messlinger K (2013). The calcitonin gene-related peptide receptor antagonist MK-8825 decreases spinal trigeminal activity during nitroglycerin infusion. J Headache Pain.

[117] Vila-Pueyo M, Strother L, Page K, Loaraine H, Kovalchin J, Goadsby PJ, Holland PR (2016). Lasmiditan inhibits trigeminovascular nociceptive transmission. Cephalalgia.

[118] Akerman S, Simon B, Romero-Reyes M (2017). Vagus nerve stimulation suppresses acute noxious activation of trigeminocervical neurons in animal models of primary headache. Neurobiol Dis.

[119] Bloom FE (1974). To spritz or not to spritz: the doubtful value of aimless iontophoresis. Life Sci.

[120] Lambert GA (1992). The spinal cord processing of input from the superior sagittal sinus: pathway and modulation by ergot alkaloids. Brain Res.

[121] Donaldson C (2002). The role of 5-HT1B and 5-HT1D receptors in the selective inhibitory effect of naratriptan on trigeminovascular neurons. Neuropharmacology.

[122] Lambert GA (2002). Suppression by eletriptan of the activation of trigeminovascular sensory neurons by glyceryl trinitrate. Brain Res.

[123] Storer RJ, Goadsby PJ (1997). Microiontophoretic application of serotonin (5HT)1B/1D agonists inhibits trigeminal cell firing in the cat. Brain.

[124] Storer RJ, Akerman S, Goadsby PJ (2004). Calcitonin gene-related peptide (CGRP) modulates nociceptive trigeminovascular transmission in the cat. Br J Pharmacol.

[125] Thankachan S (2019). Thalamic Reticular Nucleus Parvalbumin Neurons Regulate Sleep Spindles and Electrophysiological Aspects of Schizophrenia in Mice. Sci Rep.

[126] Bullitt E (1990). Expression of c-fos-like protein as a marker for neuronal activity following noxious stimulation in the rat. J Comp Neurol.

[127] Chiu R (1988). The c-Fos protein interacts with c-Jun/AP-1 to stimulate transcription of AP-1 responsive genes. Cell.

[128] Gallo FT (2018). Immediate Early Genes, Memory and Psychiatric Disorders: Focus on c-Fos, Egr1 and Arc. Front Behav Neurosci.

[129] Coggeshall RE (2005). Fos, nociception and the dorsal horn. Prog Neurobiol.

[130] Sundquist SJ, Nisenbaum LK (2005). Fast Fos: rapid protocols for single- and double-labeling c-Fos immunohistochemistry in fresh frozen brain sections. J Neurosci Methods.

[131] Morgan JI, Curran T (1988). Calcium as a modulator of the immediate-early gene cascade in neurons. Cell Calcium.

[132] Hunt SP, Pini A, Evan G (1987). Induction of c-fos-like protein in spinal cord neurons following sensory stimulation. Nature.

[133] Harris JA (1998). Using c-fos as a neural marker of pain. Brain Res Bull.

[134] Hoskin KL, Bulmer DC, Goadsby PJ (1999). Fos expression in the trigeminocervical complex of the cat after stimulation of the superior sagittal sinus is reduced by L-NAME. Neurosci Lett.

[135] Strassman AM, Mineta Y, Vos BP (1994). Distribution of fos-like immunoreactivity in the medullary and upper cervical dorsal horn produced by stimulation of dural blood vessels in the rat. J Neurosci.

[136] Sugimoto T (1998). c-fos induction in the subnucleus oralis following trigeminal nerve stimulation. Brain Res.

[137] Goadsby PJ, Zagami AS (1991). Stimulation of the superior sagittal sinus increases metabolic activity and blood flow in certain regions of the brainstem and upper cervical spinal cord of the cat. Brain.

[138] Kaube H, Hoskin KL, Goadsby PJ (1992). Activation of the trigeminovascular system by mechanical distension of the superior sagittal sinus in the cat. Cephalalgia.

[139] Strassman AM (1993). Fos-like immunoreactivity in the superficial medullary dorsal horn induced by noxious and innocuous thermal stimulation of facial skin in the rat. J Neurophysiol.

[140] Hoskin KL, Goadsby PJ (1998). Comparison of more and less lipophilic serotonin (5HT1B/1D) agonists in a model of trigeminovascular nociception in cat. Exp Neurol.

[141] Shepheard SL (1995). Comparison of the effects of sumatriptan and the NK1 antagonist CP-99,994 on plasma extravasation in Dura mater and c-fos mRNA expression in trigeminal nucleus caudalis of rats. Neuropharmacology.

[142] Nelson DL (2010). Preclinical pharmacological profile of the selective 5-HT1F receptor agonist lasmiditan. Cephalalgia.

[143] Hoskin KL (2001). Fos expression in the midbrain periaqueductal grey after trigeminovascular stimulation. J Anat.

[144] Keay KA, Bandler R (1998). Vascular head pain selectively activates ventrolateral periaqueductal gray in the cat. Neurosci Lett.

[145] Tassorelli C, Joseph SA (1995). Systemic nitroglycerin induces Fos immunoreactivity in brainstem and forebrain structures of the rat. Brain Res.

[146] Park J (2014). Differential trigeminovascular nociceptive responses in the thalamus in the familial hemiplegic migraine 1 knock-in mouse: A Fos protein study. Neurobiol Dis.

[147] Tepe N (2015). The thalamic reticular nucleus is activated by cortical spreading depression in freely moving rats: prevention by acute valproate administration. European J Neurosci.

[148] May A, Goadsby PJ (2001). Substance P receptor antagonists in the therapy of migraine. Expert Opin Investig Drugs.

[149] Polley JS (1997). The activity of GR205171, a potent non-peptide tachykinin NK1 receptor antagonist, in the trigeminovascular system. Regul Pept.

[150] Widmann C (1999). Mitogen-activated protein kinase: conservation of a three-kinase module from yeast to human. Physiol Rev.

[151] Martins-Oliveira M (2017). Neuroendocrine signaling modulates specific neural networks relevant to migraine. Neurobiol Dis.

[152] Gao YJ, Ji RR (2009). c-Fos and pERK, which is a better marker for neuronal activation and central sensitization after noxious stimulation and tissue injury?. Open Pain J.

[153] Burstein R (2000). An association between migraine and cutaneous allodynia. Ann Neurol.

[154] Oshinsky ML (2006). Insights from experimental studies into allodynia and its treatment. Curr Pain Headache Rep.

[155] Tfelt-Hansen P, Lous I, Olesen J (1981). Prevalence and significance of muscle tenderness during common migraine attacks. Headache.

[156] Burstein R (2010). Thalamic sensitization transforms localized pain into widespread allodynia. Ann Neurol.

[157] Chaplan SR (1994). Quantitative assessment of tactile allodynia in the rat paw. J Neurosci Methods.

[158] Dixon WJ (1980). Efficient analysis of experimental observations. Annu Rev Pharmacol Toxicol.

[159] Minett MS, Quick K, Wood JN (2011). Behavioral Measures of Pain Thresholds. Curr Protoc Mouse Biol.

[160] Kim SH, Chung JM (1992). An experimental model for peripheral neuropathy produced by segmental spinal nerve ligation in the rat. Pain.

[161] Hansen JM (2008). Familial hemiplegic migraine type 1 shows no hypersensitivity to nitric oxide. Cephalalgia.

[162] Tipton AF (2016). The effects of acute and preventive migraine therapies in a mouse model of chronic migraine. Cephalalgia.

[163] Farajdokht F (2018). Ghrelin attenuated hyperalgesia induced by chronic nitroglycerin: CGRP and TRPV1 as targets for migraine management. Cephalalgia.

[164] Greco R (2018). Chronic and intermittent administration of systemic nitroglycerin in the rat induces an increase in the gene expression of CGRP in central areas: potential contribution to pain processing. J Headache Pain.

[165] Ben Aissa M (2018). Soluble guanylyl cyclase is a critical regulator of migraine-associated pain. Cephalalgia.

[166] Marquez de Prado B, Hammond DL, Russo AF (2009). Genetic enhancement of calcitonin gene-related Peptide-induced central sensitization to mechanical stimuli in mice. J Pain.

[167] Yan J (2012). Sensitization of dural afferents underlies migraine-related behavior following meningeal application of interleukin-6 (IL-6). Mol Pain.

[168] Fioravanti B (2011). Evaluation of cutaneous allodynia following induction of cortical spreading depression in freely moving rats. Cephalalgia.

[169] Filiz A (2017). CGRP receptor antagonist MK-8825 attenuates cortical spreading depression induced pain behavior. Cephalalgia.

[170] Choi Y (1994). Behavioral signs of ongoing pain and cold allodynia in a rat model of neuropathic pain. Pain.

[171] Colburn RW (2007). Attenuated cold sensitivity in TRPM8 null mice. Neuron.

[172] Hargreaves K (1988). A new and sensitive method for measuring thermal nociception in cutaneous hyperalgesia. Pain.

[173] Akcali D (2017). Nitroglycerin challenge induces lateralized headache in nasociliarynerve-ligated rats: implications for chronic migraine. Turk J Med Sci.

[174] Kim SJ (2018). Differential Development of Facial and Hind Paw Allodynia in a Nitroglycerin-Induced Mouse Model of Chronic Migraine: Role of Capsaicin Sensitive Primary Afferents. Biol Pharm Bull.

[175] Anderson EM et al (2013) Use of the Operant Orofacial Pain Assessment Device (OPAD) to measure changes in nociceptive behavior. J Vis Exp (76):50336. doi: 10.3791/50336 10.3791/50336 PMC3727247 23792907

[176] Rohrs EL (2015). A novel operant-based behavioral assay of mechanical allodynia in the orofacial region of rats. J Neurosci Methods.

[177] Sureda Gibert P, Goadsby PJ, Holland PR (2018). Characterisation of an orofacial pain assessment device (OPAD) to measure facial allodynia. Cephalalgia.

[178] Pradhan AA (2014). delta-Opioid receptor agonists inhibit migraine-related hyperalgesia, aversive state and cortical spreading depression in mice. Br J Pharmacol.

[179] Deuis JR, Dvorakova LS, Vetter I (2017). Methods used to evaluate pain behaviors in rodents. Front Mol Neurosci.

[180] Akcali D (2010). Does single cortical spreading depression elicit pain behaviour in freely moving rats?. Cephalalgia.

[181] Houben T (2016). Optogenetic induction of cortical spreading depression in anesthetized and freely behaving mice. J Cereb Blood Flow Metab.

[182] Melo-Carrillo A, Lopez-Avila A (2013). A chronic animal model of migraine, induced by repeated meningeal nociception, characterized by a behavioral and pharmacological approach. Cephalalgia.

[183] de Boer I, van den Maagdenberg A, Terwindt GM (2019). Advance in genetics of migraine. Curr Opin Neurol.

[184] Lafreniere RG (2010). A dominant-negative mutation in the TRESK potassium channel is linked to familial migraine with aura. Nat Med.

[185] Royal P (2019). Migraine-Associated TRESK Mutations Increase Neuronal Excitability through Alternative Translation Initiation and Inhibition of TREK. Neuron.

[186] van den Maagdenberg AM (2004). A Cacna1a knockin migraine mouse model with increased susceptibility to cortical spreading depression. Neuron.

[187] Hall B (2018). Genome Editing in Mice Using CRISPR/Cas9 Technology. Curr Protoc Cell Biol.

[188] Recober A (2010). Induction of multiple photophobic behaviors in a transgenic mouse sensitized to CGRP. Neuropharmacology.

[189] Russo AF (2009). A Potential Preclinical Migraine Model: CGRP-Sensitized Mice. Mol Cell Pharmacol.

[190] Oshinsky ML (2012). Spontaneous trigeminal allodynia in rats: a model of primary headache. Headache.

[191] Munro G (2018). A unique inbred rat strain with sustained cephalic hypersensitivity as a model of chronic migraine-like pain. Sci Rep.

[192] Houben T (2017). Optogenetic induction of cortical spreading depression in anesthetized and freely behaving mice. J Cereb Blood Flow Metab.

[193] Zhang X (2011). Activation of central trigeminovascular neurons by cortical spreading depression. Ann Neurol.

[194] Burstein R, Cliffer KD, Giesler GJ (1987). Direct Somatosensory Projections from the Spinal-Cord to the Hypothalamus and Telencephalon. J Neurosci.

[195] Burstein R, Cliffer KD, Giesler GJ (1990). Cells of Origin of the Spinohypothalamic Tract in the Rat. J Comp Neurol.

[196] Noseda R (2010). A neural mechanism for exacerbation of headache by light. Nat Neurosci.

[197] Noseda R (2017). Neural mechanism for hypothalamic-mediated autonomic responses to light during migraine. Proc Natl Acad Sci U S A.

[198] Kim EJ (2016). Improved Monosynaptic Neural Circuit Tracing Using Engineered Rabies Virus Glycoproteins. Cell Reports.

